# Exploring the Educational Needs of Patients' Families Nearing Discharge From Intensive Care Units: A Qualitative Study

**DOI:** 10.1155/nrp/2994944

**Published:** 2025-11-25

**Authors:** Azar Darvishpour, Shiva Mahdavi Fashtami, Sepideh Babaeipour, Elham Ansari

**Affiliations:** ^1^Social Determinants of Health Research Center, Trauma Institute, Guilan University of Medical Sciences, Rasht, Iran; ^2^Department of Nursing, Zeyinab (P.B.U.H) School of Nursing and Midwifery, Guilan University of Medical Sciences, Rasht, Iran; ^3^Pirouz Hospital, Guilan University of Medical Sciences, Rasht, Iran

**Keywords:** discharge, educational need, hospital, intensive care unit (ICU), nurse, patient's family

## Abstract

**Background:**

Families of patients transitioning from the intensive care unit (ICU) to home care encounter numerous challenges. Nurses play a pivotal role in supporting these families, and identifying their educational needs is crucial for optimal patient care postdischarge. This study aimed to explore the educational needs of patients' families on the eve of discharge from special care units.

**Materials and Methods:**

A qualitative study was conducted using purposive sampling, involving 17 families of patients nearing discharge from ICUs in hospitals in eastern Guilan, Iran. Semistructured and in-depth interviews were conducted until data saturation. Data were analyzed using conventional content analysis, guided by the Graneheim and Lundman (2004) model, with MAXQDA 2007 software.

**Results:**

Data analysis resulted in the identification of 48 initial codes, which were categorized into three main categories named “Care in the shadow of fear and worry” with two subcategories (feeling worried and fear of providing independent patient care), “Perceived need for ongoing education” with two subcategories (concerns regarding complex/technical care procedures and needs for education on daily care and well-being management), and “Family evaluations of training: satisfaction and perceived adequacy” with two subcategories (satisfaction with training and dissatisfaction with the adequacy of training).

**Conclusions:**

This study highlights the significant emotional burden and worries experienced by family members as they prepare for their patient's discharge from the ICU. Healthcare providers must address these concerns and offer ongoing education and support to enhance home care safety and competence.

## 1. Background

Each year, thousands of patients are admitted to the intensive care unit (ICU), where their families often face challenging and stressful situations, including adapting to a new environment, dealing with uncertain outcomes, and, in some cases, the potential loss of their loved ones [1]. In the ICU, patients are typically unable to make decisions regarding their care, placing this critical responsibility on their family members, a role that can be overwhelming [2]. Consequently, family support is a vital component of patient care and an essential element in the treatment process that warrants significant attention [3]. To ensure comprehensive and high-quality nursing care, ICU nurses must address not only the needs of the patients but also those of their families [4]. This dual focus is crucial for enhancing the overall care experience and improving patient outcomes.

The transition of patients from the hospital to home care is a critical phase that can lead to numerous problems and an increased rate of patient complications if not managed effectively [5]. These patients may be discharged with necessary medical devices such as tracheostomies, gastric tubes, and Foley catheters, which place a significant burden on caregivers and necessitate ongoing support from a multidisciplinary team of home care experts [6]. Consequently, the lack of familiarity among family members with the appropriate type and care method can lead to numerous problems and an increased rate of patient complications [7]. In the transition of patients from the hospital to the home environment, a structured and continuous care program is required, in which nurses play a crucial role as coordinators to ensure safe and high-quality care with the active participation of the family [8]. However, family members may struggle to identify their own needs, and nurses may also find it challenging to anticipate these needs accurately [7].

The lack of structured educational programs has led caregivers to seek information from unreliable sources [5]. This underscores the critical role of nurses as professional healthcare providers. Nurses play a pivotal role in disseminating accurate information and fostering healthy behaviors through family counseling [4]. Nursing staff should begin preparing family caregivers for discharge from the moment of patient admission, encompassing planning, family preparation, and developing an individualized treatment plan for the patient [9]. A scientific assessment of family needs is the first step in preventing unintentional neglect of patient and family care [10]. Such assessments provide valuable insights that enhance the understanding, satisfaction, and decision-making capabilities of caregiver families [11].

Several studies have explored the educational needs of families as patients approach discharge from ICUs. For instance, Alsharari et al. found that the highest need was for reassurance and information [12]. Padilla Fortunatti's study highlighted the importance of hope for favorable outcomes and a sincere relationship with the healthcare staff [13]. Mitchell et al. identified personal distress and its adjustment, along with guidance and care, as the most significant needs [14]. Sarhadi et al. reported that families primarily required assurance of the quality and quantity of patient care, information about the treatment process, and postdischarge education [11].

Given the variations in needs across different regions and the paucity of research on this topic in Iran, there is a pressing need for more comprehensive knowledge regarding the needs and preferences of families of patients hospitalized in special wards. Therefore, recognizing the importance of this issue and the growing acknowledgment that families are integral to the treatment and health process of patients in special wards [15], researchers aimed to explore the educational needs of these families. On the other hand, qualitative research is designed to gain a deep understanding of patients' values, needs, and preferences. The inductive approach in qualitative research allows findings to emerge from raw data without imposing pre-existing assumptions on the environment under study [16]. Given that the educational needs of individuals are multidimensional and influenced by multiple factors, a qualitative approach and in-depth study of individuals who consistently face various challenges are essential. A literature review indicates that, despite the research conducted on patients hospitalized in ICUs and their families, no qualitative study has specifically examined the educational needs of these patients' families. Therefore, the present study was conducted to fill this knowledge gap and to identify the educational needs of families of patients on the verge of discharge from ICUs in hospitals in eastern Guilan.

## 2. Materials and Methods

### 2.1. Study Design, Setting, and Participants

This qualitative study was conducted in 2024, focusing on the families of patients hospitalized in the ICUs of hospitals located in eastern Guilan, Iran. The participants in this study comprised family members (including children, parents, and siblings) who were designated as the primary caregivers for the patients following their discharge from the hospital. The sampling method was purposive, ensuring that participants met specific inclusion criteria. These criteria included having a familial relationship with the patient, the capacity and responsibility to care for the patient postdischarge, the ability to communicate effectively, literacy, and a willingness to participate in the study. The primary exclusion criterion was the participant's unwillingness to continue participation throughout the study duration.

### 2.2. Research Instruments

Data collection was achieved through semistructured, in-depth interviews. Participants who met the inclusion criteria were provided with detailed information about the study, and their consent for participation was obtained. The time and location of the interviews were scheduled according to the participants' convenience, and confidentiality was assured. To guarantee confidentiality, all participants were assigned identification codes instead of names in the transcripts, and any potentially identifying details were removed during transcription. Audio files and transcripts were stored in password-protected electronic folders accessible only to the research team. Findings were reported in aggregate form without linking quotations to personal identifiers. Data collection proceeded until data saturation was achieved, indicating that no new information was being obtained from subsequent interviews [17]. In total, 20 interviews were conducted with family members. Data saturation was reached after 17 interviews; however, three additional interviews were carried out to ensure completeness and confirm saturation. Each interview was conducted individually and face-to-face, utilizing a set of guiding questions.

The interview guide was developed after a comprehensive review of the relevant literature on family educational needs in ICUs and postdischarge care, as well as consultation with three faculty experts in critical care nursing and qualitative research. Additionally, principles from recent studies on structured guidance, interactive engagement, and informal learning were incorporated to enhance participant understanding and confidence [18, 19]. The guide included a brief demographic section (e.g., age, gender, marital status, education level, employment status, and relationship to the patient) and was organized around four main thematic areas: (a) perceived educational needs for home care, (b) concerns and challenges regarding the patient's transition from hospital to home, (c) expectations from and perceived gaps in support by healthcare providers during discharge, and (d) preferred learning modalities and potential barriers to receiving education.

Sample demographic items included: “What is your age?”, “What is your highest level of education?”, and “What is your relationship to the patient?”. Sample core open-ended questions included: “What do you think you need to know to care for your loved one at home?”, “What concerns do you have regarding the care of your loved one after discharge?”, “How can healthcare providers support you in preparing for discharge?”.

The researcher tried to have minimal interference in the interview process. However, leading and clarifying questions such as “Could you elaborate on that?”, and “Do you mean…?” were used to guide the conversation and ensure that the interview objectives were met. The interviews were recorded using a digital voice recorder, with the participants' permission. Additionally, field notes were taken during the interviews to capture nonverbal cues and contextual information.

### 2.3. Data Analysis

For the data analysis in this study, the conventional content analysis method, based on the model proposed by Graneheim and Lundman [20], was employed. Content analysis is one of the valid research methods for data analysis. This type of analysis is a systematic approach that, by coding and classifying data, can be used to discover large amounts of textual information to determine the trends and patterns of words, their relationships, structures, and communication discourses [21].

In this study, the entire transcript of each interview was considered as the unit of analysis. Following the completion of each interview, the recorded statements were listened to multiple times and transcribed verbatim. The transcriptions were then converted into text files. Each participant's interview text was read and reviewed several times to gain an overall understanding of the data. Subsequently, important sentences pertinent to the phenomenon under study were identified and underlined, forming the initial meaning units. These meaning units were then condensed, and each significant phrase was assigned a code. The codes were summarized and categorized into subcategories. Finally, the subcategories were synthesized to form the main categories, based on their similarities and differences. MAXQDA 2007 software was used to facilitate the management of codes, organization of meaning units, and retrieval of text segments. The software served as a supporting tool to systematize data storage and categorization; however, the processes of interpretation and thematic analysis were performed manually by the research team.  Table 1 presents an example of the meaning units, initial codes, and subcategories of the first main category (Care in the Shadow of Fear and Worry).

### 2.4. Rigor

To ensure the rigor in this study, the trustworthiness criteria proposed by Lincoln and Guba [22] were applied. These criteria include credibility, dependability, confirmability, and transferability. To meet these criteria, the researchers performed the following:

1. Credibility: carefully selected participants and maintained long-term engagement with them to build trust. Sufficient time was allocated to address all questions and concerns.
2. Dependability: continuously reviewed and compared data and categories for similarities and differences, ensuring the consistency and reliability of the analysis. In addition, all initial and focused codes were independently reviewed by the researchers. Any discrepancies were discussed and resolved, and a detailed audit trail of coding decisions was maintained in MAXQDA to document the analytic process.
3. Confirmability: rechecked findings with participants and submitted the coding framework and extracted categories for external audit. The audit was conducted by two faculty members specializing in qualitative research and critical care nursing who were not directly involved in the study. They independently reviewed a sample of transcripts, verified the consistency between raw data, codes, and categories, and provided feedback on the analytic process. Any suggestions or discrepancies raised by these experts were discussed with the research team and incorporated as appropriate.
4. Transferability: provided detailed and rich descriptions of the research process and findings, enabling readers to understand and apply the results to other contexts.

### 2.5. Ethical and Research Approvals With the Date

The study was approved by the Ethics Committee at the Guilan University of Medical Sciences (ID IR.GUMS.REC.1403.021). Participation in the interview was completely voluntary. The participants were informed that the interview would be recorded and that the data collected would not be disclosed. Written informed consent was obtained from participants. They were assured that they could withdraw from the research whenever they wished, and all their information would remain confidential.

## 3. Results

In this study, a total of 17 families of patients hospitalized in ICU wards participated. The demographic characteristics of participants are presented in Table 1.

The majority of the participants were female (64.7%). In terms of marital status, the majority were married, and in terms of literacy, most of them were below the diploma level.

Data analysis regarding the educational needs of families of patients on the verge of discharge from ICUs from the perspective of the patients' companions led to the emergence of 48 initial codes and three main categories named “Care in the shadow of fear and worry” with two subcategories (feeling worried and fear of providing independent patient care), “Perceived need for ongoing education” with two subcategories (concerns regarding complex/technical care procedures, and most basic care education needs), and “Family evaluations of training: satisfaction and perceived adequacy” with two subcategories (satisfaction with training and dissatisfaction with the adequacy of training), which are discussed below.  Table 2 presents the meaning units, initial codes, and subcategories of the first main category (care in the shadow of fear and worry).

### 3.1. Care in the Shadow of Fear and Worry

This category reflects the emotional turmoil that families experience in the complexities of caring for their patients. The findings showed that the experiences of family members who were waiting to take on the responsibility of caring for their loved ones at home were accompanied by feelings of fear and worry. Two subcategories under this main category were: feeling worried and fear of providing independent patient care.

#### 3.1.1. Feeling Worried

This subcategory indicated that family members of ICU patients on the verge of discharge had deep concerns about their ability to provide adequate care for their loved ones. This concern was multifaceted and stemmed from a variety of factors, including perceived incompetence, limited experience, and uncertainty about the patient's condition. Some participants expressed concerns about their inexperience in caring for the patient, especially in the home setting, and were concerned about being unable to provide adequate care. Participant 5 stated “*Our concern is lack of experience. Because we do not have the experience of caring for the patient. We are worried that we will not leave less for our patient”.*

Lack of literacy and perceived inability to manage the patient's care at home were raised as concerns by some family members. Participant 4 stated “*My concern is how to care for the patient at home. I feel like I cannot do it. Because I am not literat*e.”

Additionally, the patient's condition also contributed to family members' concerns, with some expressing worries about the patient's unstable condition and the possibility of complications after discharge. The uncertainty and unpredictability of the patient's condition created a sense of discomfort and anxiety among family members. Some family members were concerned about the patient's level of consciousness and its impact on their care, as well as the uncertainty about the patient's future. Participants 3 and 7 stated “*We were concerned about the patient's level of consciousness*” and “*I want to know what will happen in the future? I am worried that his level of consciousness will not increase and improve*.”

These concerns were exacerbated by the need to manage specific and complex care procedures, such as administering medication, changing positions to prevent pressure ulcers, suctioning, feeding, and monitoring blood pressure, which family members felt unprepared to perform. Participant 6 said “*My concern is the inability to provide care. I did not think my patient would be discharged in this condition. The correct way to feed, whether to feed quickly or slowly, monitoring blood pressure, and performing suctioning were my main concerns*.” Participant 8 stated “*My main concern is suction, how to give medication, changing positions to prevent bedsores*.”

The need for education to reduce these concerns was a theme that participants mentioned, as family members felt unprepared to care for their loved ones with complex needs. Participant 12 stated “*We need education. I feel like if we take the patient home with this special condition and reduced consciousness and I am unable to care for them, what should I do?”.*

#### 3.1.2. Fear of Providing Independent Patient Care

In this subcategory, families expressed concerns about taking on this responsibility independently, in addition to concerns about their ability to care for their patients. This fear was mainly due to the patient's condition and the stressful circumstances of the patient, as well as the family's perceived lack of knowledge and skills to effectively handle these situations. Some family members stated that they had generally learned to care for their loved ones, but this was accompanied by fear and stress, and they were constantly concerned about their ability to provide adequate care. Participant 7 stated “*We learned to care, but it was accompanied by fear and stress. Because we were always worried that our loved one would get sick and we would not be able to care for him or her properly.*” Others felt that they were afraid of caring because their patient was not in a normal condition. This highlighted the need for appropriate support and training. Participant 8 stated *“I was afraid of caring for my patient alone. Because our patient was not in a normal condition*.”

In particular, some family members were concerned about managing complex care procedures, such as suctioning and clearing phlegm, which caused them significant stress and anxiety.

Participant 13 stated “*We were stressed and afraid, especially regarding coughing during suction and phlegm in the throat. Once we called 115 emergency, they came and taught us and said that these conditions are normal.*” This fear and anxiety led some family members to seek additional help and support, such as hiring a home nurse to help care for their patients. Participant 1 stated “*I felt I needed training. I was afraid to care alone. I was too stressed that I would not be able to care for my patient. To reduce the stress of caregiving, I hired a nurse to help us at home*.”

In addition, family members expressed a strong need for training to reduce their fear and anxiety about caring for their loved ones independently. Participant 2 stated “*I was very stressed about caring for my patient. I was afraid to care alone because I felt like I didn't know enough*.” This highlights the importance of providing comprehensive education and support to family members to empower them to care for their loved ones with confidence.

### 3.2. Perceived Need for Ongoing Education

Feeling the need for continuing education was raised as one of the important concerns of families of patients on the verge of discharge from the ICU. This main category included two subcategories: “Concerns regarding complex/technical care procedures” and “Needs for education on daily care and well-being management”.

#### 3.2.1. Concerns Regarding Complex/Technical Care Procedures

The findings showed that family members of patients on the verge of discharge from the ICU need education on various aspects of patient care. Some family members reported that they had received training from nurses during their loved one's stay in the ICU, but they still felt unsure about performing some specific care procedures. For example, Participant 4 stated “*The nurses taught us how to feed the patient, but I didn't understand*.” Similarly, Participant 2 reported “*I received most of the care education in the ICU, but I still feel that I need education*.” However, despite receiving training, some family members expressed concerns about their ability to perform specific care tasks, such as suctioning and tube feeding. They also acknowledged that mastering these skills requires time, practice, and ongoing support. Other family members reported that the specialized medical terminology was a significant challenge in understanding care instructions for them. Participant 7 stated “*They taught me twice. They told me a few models but I didn't understand. The names of the food were in English. One of the nurses explained it in simple language, which was good and I understood*.” This highlights the importance of communication and simple language when providing training to family members.

In particular, some family members expressed concerns about suctioning, tracheostomy care, and airway management. Participant 14 stated “*I still need training. Especially about how to feed and keep the patient's throat (airway) open*.” Participant 9 stated “*They trained us and gave us feedback on how to suction, tube feeding, and care to prevent bedsores.*” Participant 5 stated *“At first, my biggest concern was the suctioning of the patient until it gradually became normal for me*.” Participant 2 also stated “*Tracheostomy care, how to suction, and dealing with the patient's cough during suction were things I felt I needed training on*.” In addition, some family members expressed fear and anxiety about performing specific care tasks such as suctioning. Participant 12 stated “*Suctioning was the most daunting task for me. I was afraid to do it.”* This highlights the need for further training in implementing specialized procedures to reduce fear and anxiety about caring for relatives.

#### 3.2.2. Needs for Education on Daily Care and Well-Being Management

The results showed that family members of patients on the verge of ICU discharge were highly interested in education regarding various aspects of patient care. Despite receiving education before discharge from the ICU, family members still felt that they needed more education to care for their loved ones. Some family members reported that they wanted to learn more about specific care procedures such as suctioning, tube feeding, and pressure ulcer prevention. Participant 1 stated “*I was given education on suctioning, gavage, and how to care for the patient before discharge from the ICU, but I feel that I still need education.*” Participant 4 stated “*Education on how to perform suctioning, signs of infection and illness, and the ventilator are some of the things I still want to learn.”* Family members also expressed a desire to learn more about the patient's nutritional needs, including tube feeding and nutritional supplements. Participant 7 stated *“I want to learn and know more about things like tube feeding (what to give the patient, types of nutritional supplements, powdered foods).*” In addition, family members wanted to learn more about wound care, preventing bedsores, changing dressings, changing the patient's position, bathing in bed, doing physical therapy, and exercising.

### 3.3. Family Evaluations of Training: Satisfaction and Perceived Adequacy

This category highlighted a complex spectrum of satisfaction and dissatisfaction with the educational training provided to the patients' families. This category was composed of two subcategories: “satisfaction with the training” and “inadequacy of training”.

#### 3.3.1. Satisfaction With Training

This subcategory indicates that some family members of patients on the verge of discharge from the ICU expressed satisfaction with the training they received. Some family members of patients reported that the training was practical and informative and helped them increase their skills and confidence in caring for their loved ones. Some participants reported that they were satisfied with the training they received from the nurses, referring to the practical nature of the training and the learning opportunities they had in the ward. Participant number 12 stated *I was satisfied with the training, they gave us practical training and I learned. This predischarge training was really good. It made me learn how to care for the patient. I am now able to do things myself*.” Similarly, Participant 2 reported that “*The level of training was excellent. My daughter learned how to use the ventilator. They (nurses) held a training class for us inside the ICU*.”

Some participants reported that while they were satisfied with the training, they also had some concerns and apprehensions that prevented them from receiving effective training, which was of course managed by training other family members. Participant 4 stated “*The level of training was good, but because I was apprehensive. I sent other family members to learn the training and I later learned from them.”*

In addition, some family members reported that the training was well structured and that the nurses were effective teachers. Participant 5 stated “*The training in the ICU was very good. The nurses were good at teaching. I took notes and asked them again if I didn't understand something. Sometimes I called and they helped.*” This suggests that for effective training, nurses need to be available to provide ongoing support and guidance.

In addition, some family members reported that the training was well structured and that the nurses were effective teachers. Participant 5 stated “*The training in the ICU was very good. The nurses were good at teaching. I took notes and whatever I didn't know, I would ask them again. Sometimes I would call and they would help.*” This suggests that for effective training, nurses need to be available to provide ongoing support and guidance.

#### 3.3.2. Dissatisfaction With the Adequacy of Training

This subcategory showed that some family members of patients on the verge of discharge from the ICU expressed dissatisfaction with the training they received. They reported that the training was inadequate and did not meet their needs. Some family members reported that they did not learn everything they needed to know, citing specific areas such as feeding, bathing, and wound care. Participant 4 stated “*I haven't been trained on everything yet. What to feed and how to bathe the patient. The training was short. I think it needs to be repeated several times*.” Other family members also reported that the training was too short and they did not have enough practice. They felt that the brief nature of the training did not provide them with enough knowledge and skills to effectively care for their loved ones. Participant 5 stated “*The training was short and we didn't have enough practice. We were worried that we wouldn't be able to cope. The hospital staff said we could use home care centers.*”

Some family members also reported that the training was not tailored to their individual needs and learning styles. Participant 14 stated “*The training and care are not familiar to us and are very daunting. Sometimes the training was fast-paced because the ward was crowded and I could not learn well*”.

Some participants suggested that the training should be longer and more comprehensive so that they could better learn the training. They also expressed their desire for more practical training and an opportunity to practice the skills they were being taught. In addition, they requested written materials such as training and brochures to supplement the verbal instructions and provide a reference for their future use. Some participants also suggested that all family members (not just one person) should be trained and their questions answered. In addition, they suggested that educational materials, such as CDs or videos, be provided to them before discharge to facilitate learning and better retention. Several participants also suggested that they be allowed to videotape the training so that they could later watch it and review the training received. Some family members reported that they preferred more interactive and engaging training methods such as note-taking and recording. Participant 13 stated “*The training was not enough. I didn't learn. I like to write it down, explain it, then I do it so that I fully learn. To learn better, we need to take notes. It would be better if they allowed us to videotape and record the training*.”

## 4. Discussion

This study elucidates the educational needs of patients' families nearing discharge from the ICU. Three primary categories emerged: “Care in the shadow of fear and worry”, “Perceived need for ongoing education”, and “Family evaluations of training: satisfaction and perceived adequacy.”

In the first category “Care in the shadow of fear and worry,” family members of patients nearing ICU discharge expressed profound concerns about their ability to provide adequate care for their loved ones. The subcategories “feeling worried” and “fear of providing independent patient care” underscore the intricate relationship between fear, worry, and perceived capability. These findings indicate that family members of ICU patients have significant apprehensions regarding their caregiving abilities, which are influenced by various factors such as personal limitations, lack of experience, and uncertainty about the patient's condition. This pervasive fear is not merely anxiety; it represents a fundamental crisis of confidence. When families doubt their ability to perform life-sustaining tasks like suctioning or managing a tracheostomy, it transforms the home from a place of healing into a potential site of crisis, directly impacting patient safety and family well-being. This aligns with previous research that has identified substantial challenges faced by families of ICU-discharged patients. For instance, Stayt and Venes (2018) documented high levels of psychological distress, including anxiety, depression, and post-traumatic stress disorder, among these families [23]. Similarly, Meiring-Noordstra et al. found that relatives often felt unprepared for their caregiving roles, experienced a lack of involvement in care decisions, and received limited information during the transition from the ICU to home [24].

The transition from the ICU to home is generally associated with feelings of uncertainty and a sense of burden [25]. Family caregivers face significant challenges during this transition, often feeling unprepared for their new responsibilities, particularly in medication management and complex caregiving tasks [26]. They experience emotional stress and feel overwhelmed by the extensive responsibilities they must assume [27]. The physical and emotional demands of caregiving can significantly impact their professional and social lives [23]. Family members require both formal and informal support to effectively cope with the psychological and physical challenges [25]. To effectively support families, healthcare providers should assess their educational needs and implement targeted interventions to enhance their caregiving skills and coping strategies [28]. Unmet needs can contribute to increased anxiety, which may persist even after discharge [29]. For instance, a brief psychobehavioral training program was effective in managing stress and anxiety among primary family caregivers and improving their overall satisfaction [30]. Open-visitation policies and the creation of private family suites in the ICU have also been associated with higher levels of need fulfillment and satisfaction [31]. These findings emphasize the importance of incorporating family needs into nursing education programs and implementing targeted interventions to support family members, potentially reducing their psychological burden during the ICU experience [30,32]. While open-visitation policies and private family suites in the ICU can facilitate early family involvement, it is crucial to recognize that this is just the first step in a continuum of care. To address postdischarge fear and skill deficits, interventions should focus on discharge education, including simulation training, structured predischarge teaching, postdischarge telehealth support, and caregiver competency assessments. These strategies are directly aligned with our core findings and aim to prepare families for safe and confident caregiving at home.

The second main category identified the need for ongoing education as a major concern for families of patients on the verge of ICU discharge. This category included two subcategories: “concerns regarding complex/technical care procedures” and “most basic care education needs.” Research indicates that families of patients discharged from ICUs have significant educational and support needs. Several studies have emphasized the importance of addressing the educational needs, experiences, and satisfaction of families with care. These studies have shown that families require high levels of information on various aspects of care, with self-care, nutrition, and medication management being top priorities [28, 33]. Key information needs include assurance of appropriate treatment, updates on the patient's condition, and prognosis [3].

Results from various studies have identified the most important needs expressed by families, which include receiving honest answers to questions, reassurance about the patient's care, and regular communication with healthcare providers [31, 32]. Emotional states, cognitive impairment, literacy levels, and complex medication regimens can significantly impede caregivers' ability to effectively manage medications [34]. To improve the caregiving experience, caregivers need adequate preparation, including specific knowledge and practical skills at different stages of their role [35]. Implementing family-centered care (FCC) and addressing these educational needs can enhance overall satisfaction and reduce emotional and psychological distress among family members of ICU patients [3]. Effective communication between healthcare professionals, patients, and caregivers is also crucial [36]. Caregivers require emotional and practical support, as well as education on home care techniques [27, 36]. Studies suggest that open-visit policies and the provision of dedicated spaces for families of these patients in the ICU can help address these needs more effectively [31]. To address these challenges, healthcare professionals should assess family needs, provide targeted educational interventions, and implement comprehensive follow-up programs to support families during the transition from hospital to home care [25, 28]. Additionally, to improve outcomes, healthcare professionals should focus on better discharge planning, familiarization with medications, and providing ongoing support to patients and caregivers at home [36].

The third main category highlighted a complex spectrum of satisfaction and dissatisfaction with the adequacy of training provided to families of patients on the verge of ICU discharge. This category emerged from two subcategories: “satisfaction with training” and “dissatisfaction with the adequacy of training.” Consistent with this finding, research also shows that families of patients admitted to the ICU have varying levels of satisfaction with the education and care provided. For example, studies by Ali et al. and Lam et al. found that while the overall level of satisfaction was generally positive, it was often accompanied by dissatisfaction in specific areas [37, 38].

In the study by Liyew et al., the highest scores for family satisfaction were related to the time provided to receive answers to their questions and the willingness of the intensive care staff to address these questions. Families reported receiving sufficient information to understand the patient's condition, treatment plan, and progress [39]. However, this aligns with our findings that while some families reported satisfaction with the training, many simultaneously expressed dissatisfaction due to the brevity and inconsistency of information. Specifically, our participants reported challenges in accessing comprehensive guidance and receiving consistent instructions, directly mirroring Liyew et al.'s observation that satisfaction can be undermined by poor information accessibility. This discrepancy in satisfaction levels underscores the importance of effective communication and consistent provision of information in enhancing the family experience of ICU care. Indeed, the discrepancy in satisfaction levels regarding information provision may stem from families' perception that the information they receive is incomplete or inadequate. When families feel that they lack essential information about their loved one's condition, treatment options, and prognosis, it can lead to feelings of frustration and dissatisfaction. Therefore, ensuring that families receive comprehensive and clear information is crucial to improving satisfaction levels with ICU care. To enhance the quality of care and family satisfaction, healthcare providers should prioritize effective and regular communication with family members. Keeping them informed about the patient's condition and treatment plan is essential [39]. Although educating families can increase the workload for nurses, it allows families to learn home care techniques through deeper interactions and improve their caregiving skills for home care [40]. Implementing structured educational programs and providing regular updates can help bridge the information and support gap, ultimately leading to improved outcomes for both patients and their families.

The findings of this study highlight the urgent need to improve educational support for family members of patients on the verge of discharge from the ICU. Despite the critical role that family members play in providing ongoing care and support for patients after discharge, the present study found that many family members are inadequately prepared to assume this responsibility. Specifically, family members reported not having learned essential skills such as suctioning, feeding, bathing, and wound care. This underscores the importance of tailoring educational programs to meet the unique needs and concerns of family members. This finding is consistent with previous studies that have emphasized the need for more comprehensive and targeted educational programs for family members of ICU patients [31, 32]. The educational needs of family members can vary significantly depending on factors such as the patient's specific medical condition, the family member's prior caregiving experience, and their learning style. Healthcare providers should adopt a more personalized approach to education [41] and utilize a variety of educational methods and materials to ensure that family members feel confident and competent in their ability to provide care [42]. This personalized approach may include one-on-one training sessions, interactive workshops, multimedia resources, and ongoing support through follow-up visits or telehealth services. By addressing the diverse needs of family members, healthcare providers can enhance the quality of care and improve the overall well-being of both patients and their caregivers.

Specialized skills training is a crucial aspect of educational content, as caregivers need to acquire specific skills to care for their loved ones at home. Studies have shown that caregivers go through a learning journey that begins with intensive training and progresses to expertise through experience and self-directed learning [43]. Interactive educational technologies have been effective in improving caregiver knowledge and skills, particularly in areas such as tube feeding and patient transfer [44]. Implementing educational and practical interventions is crucial to enhancing families' skills and readiness for post-ICU care, ultimately improving outcomes for patients and their caregivers [25, 28]. These studies emphasize the importance of structured educational programs and ongoing support for caregivers to ensure the continuity of care at home, highlighting the potential of technology-based learning tools in caregiver education. Furthermore, the results of this study emphasize the development of family-centered models of care in the ICU. FCC is an approach that emphasizes the vital role of families in patient care, particularly in critical care settings [45, 46]. This approach aims to empower families through education, support, and participation in decision-making. FCC has also been shown to increase the quality of care and patient satisfaction [47].

This study had several limitations. The first limitation was that the research was conducted with a qualitative design, and, given the nature of the study, the generalizability of the findings is limited. On the other hand, this study was conducted in the ICUs of hospitals in Eastern Guilan, which may not be representative of other regions or cultural contexts. Consequently, the transferability of the findings to other settings may be limited.

The second limitation was that the participants in this study were families of patients on the verge of discharge from the ICU, and therefore may not include diverse perspectives such as those of healthcare professionals or other members involved in patient care. The third limitation was that the sampling of this study was purposive, which may introduce selection bias in the selection of participants. The families participating in the study may not be representative of all families of patients on the verge of discharge from the ICU.

## 5. Conclusion

Overall, the findings of this study underscore the emotional burden that family members endure when caring for their loved ones nearing ICU discharge. The fears and concerns identified in this study highlight the need for targeted interventions to support and educate family members in their caregiving roles. By addressing these concerns, healthcare providers can help reduce the emotional distress associated with ICU care in-home caregivers and promote a more effective and supportive caregiving experience. The study also emphasizes the importance of ongoing education and support for family caregivers, including information on managing medications, using equipment, and recognizing signs of infection. Healthcare providers should prioritize these educational and support measures and consider implementing standardized programs that focus on specific patient care, medication management, and emotional support. It is recommended to develop and implement such programs, ensuring that healthcare providers receive FCC training and fostering collaboration between healthcare providers, patients, and families to facilitate the transition of patients from the hospital to home. Importantly, preparing a family for ICU discharge is not merely an educational task; it is an ethical imperative to prevent avoidable harm and transform fear into confidence at the patient's bedside.

## Figures and Tables

**Table 1:** Demographic characteristics of participants (*N* = 17).

Variable	Category	*n*	%
Gender	Female	11	64.7
	Male	6	35.3

Marital status	Married	13	76.5
	Single/divorced	4	23.5

Literacy level	Below diploma	10	58.8
	Diploma & higher	7	41.2

**Table 2:** Meaning units, primary codes, and subcategories of the first main category (care in the shadow of fear and worry).

Meaning units	Primary codes	Subcategories	Main category
“My concern is how to care for the patient at home. I feel like I cannot do it. Because I am not literate.” (Participant 4)	Concerns about care due to illiteracy and inability	Feeling worried	Care in the shadow of fear and worry
“Our concern is lack of experience. Because we do not have the experience of caring for the patient. We are worried that we will not leave less for our patient”. (Participant 5)	Concerns about lack of or little experience or the possibility of procrastination		
“ My concern was my patient's consciousness. I wanted to know what the future held for my son. I was worried that his level of consciousness would not improve and he would not recover.” (Participant 7)	Concern about the patient's state of consciousness		
“I was afraid of independent patient care because I didn't know what to expect.” (Participant 14)	Fear of independent patient care	Fear of providing independent patient care	
“My concern is the inability to provide care. I didn't think my patient would be discharged in this condition. The correct way to feed, whether to feed quickly or slowly, monitoring blood pressure, and performing suctioning were my main concerns.” (Participant 6)	Concerns about inability to care		

## Data Availability

The data that support the findings of this study are available upon request from the corresponding author. The data are not publicly available due to privacy or ethical restrictions.
